# Supplementary material for: Electronic transport driven by collective light-matter coupled states in a quantum device
Source: Nat Commun. 2023 Jul 3;14:3914. doi: 10.1038/s41467-023-39594-z (PMC10318076; doi:10.1038/s41467-023-39594-z)
Supplement: Supplementary file 1 — Supplementary Information [file 41467_2023_39594_MOESM1_ESM.pdf]

Supplementary Information

Electronic transport driven by collective light-matter coupled states in a quantum device

Francesco Pisani[1], Djamal Gacemi[1], Angela Vasanelli[1], Lianhe Li[2], Alexander Giles Davies[2], Edmund Linfield[2], Carlo Sirtori[1] and Yanko Todorov[1]**
[1]Laboratoire de Physique de l'Ecole Normale Supérieure, ENS, Paris Sciences et Lettres, CNRS, Université de Paris, 24 Rue Lhomond, 75005 Paris, France
[2]School of Electronic and Electrical Engineering, University of Leeds, Leeds LS2 9JT, United Kingdom

* Corresponding Authors: Francesco Pisani - francesco.pisani@phys.ens.fr; Yanko Todorov yanko.todorov@phys.ens.fr.

Supplementary Note 1: Quantum Model for Infrared detectors in the ultra-strong coupling regime

1.1. General model

All the parameters of the quantum model are summarized in Figure S1. As explained in the main text, we first define a quantum Hamiltonian which couples the detector region to a single mode microcavity. We then use input-output formalism and density matrix approach to express the photocurrent generated under incident IR radiation.

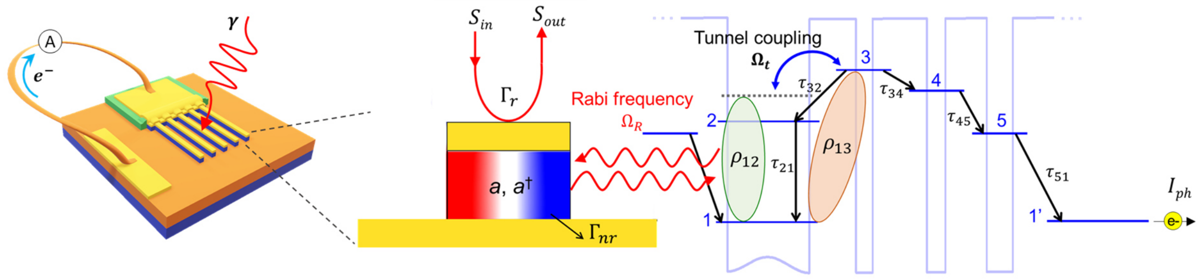

Figure S1. Diagram describing the quantum model for infrared photodetector in the ultra-strong light-matter coupling regime. A quantum cascade detector is coupled with a single mode of a patch microcavity through the quantum Hamiltonian (1-3). Our aim is to determine the photocurrent I_{ph} induced by an external photon drive S_{in}. The two ellipses represent the quantum coherences ρ_{21} and ρ_{31} which are particularly important for our quantum theory. The scheme of the photodetector highlights the grating coupling the light into the active region.

The full microscopic Hamiltonian is written as follows:

(1) $$\hat{H} = \hat{H}_{cavity} + \hat{H}_{electrons} + \frac{(\hbar\omega_{P1})^2}{4E_{21}}\hat{P}_{12}^2 + i\hbar\Omega_{R1}\sqrt{E_{cK=1}/E_{21}}(a^\dagger - a)\hat{P}_{12} + \hbar\Omega_t \sum_{\mathbf{k}}\left(c^\dagger_{2\mathbf{k}}c_{3\mathbf{k}} + c^\dagger_{3\mathbf{k}}c_{2\mathbf{k}}\right),$$

(2) $$\hat{H}_{cavity} = E_{cK=1}\left(a^\dagger a + 1/2\right)$$

(3) $\hat{H}_{electrons} = \sum_{\mathbf{k}i} \hbar\omega_{i\mathbf{k}} c_{i\mathbf{k}}^{\dagger} c_{i\mathbf{k}}$

Here $c_{i\mathbf{k}}^{\dagger}, c_{i\mathbf{k}}$ create/destroy an electron on the subband i with an in-plane momentum $\boldsymbol{k}$ and $\hbar\omega_{i\mathbf{k}} = \hbar\omega_i + \frac{(\hbar \boldsymbol{k})^2}{2m*}$ is the single-particle energy of conduction band electrons, with m^* their effective mass (parabolic dispersion is supposed for simplicity). As stated in the main text, $\hat{P}_{12} = \sum_{\mathbf{k}} \left(c_{1\mathbf{k}}^{\dagger} c_{2\mathbf{k}} + c_{2\mathbf{k}}^{\dagger} c_{1\mathbf{k}} \right)$ is an operator that describes the collective electronic polarization between the subbands 1 and 2. The third term of the right hand side of (1) describes dipole-dipole interactions that lead to plasmonic effects and a blue-shift of the 1→2 transition[1], with $\hbar\omega_{P1}$ the plasma energy defined in such a way that:

(4) $\omega_{P1}^2 = \frac{e^2}{m*\varepsilon\varepsilon_0 S L_{QW}}$

This quantity can be regarded as the plasma frequency of a single electron (see also **2**), where S is the area of the heterostructure and L_{QW} an effective thickness of the quantum well[1]. Thus, the fourth term describes interaction between the 1→2 transition and the cavity with a single electron coupling constant $\Omega_{R1} = (\omega_{P1}/2)\sqrt{F}$ [1]. Here $F=N_p L_{QW}/L_{cav}=0.13$ expresses the geometrical overlap between the electron gas and the cavity mode, with N_p the number of quantum wells and L_{cav} the total thickness the patch cavity. The last term of (1) corresponds to the coherent tunnelling of electrons between the levels 2 and 3. In the above Hamiltonian, for simplicity, we neglect the plasmon effects other than the ones arising between transition 1→2. This is justified by the small overlap of wavefuctions i>2 with the fundamental level 1, by design. Also, the photon wavevector of the cavity photon $\sim E_{cK=1}/\hbar c$ being typically very small in comparison with the electronic wavevectors $\boldsymbol{k}$ we consider only vertical excitations, which justifies the form for $\hat{P}_{12}$.

A natural framework to express the electronic relaxation is the density matrix approach[3], where the Hamiltonian evolution is supplemented with relaxation terms. The system is considered at a temperature T=0 K, with no electron above the Fermi level in the fundamental state. To implement this approach, we define electronic populations $N_i=\sum_{\boldsymbol{k}} \langle c_{i\boldsymbol{k}}^{\dagger} c_{i\boldsymbol{k}} \rangle$ and coherences $\rho_{ij}=\sum_{\boldsymbol{k}} \langle c_{i\boldsymbol{k}}^{\dagger} c_{j\boldsymbol{k}} \rangle$. The relaxation paths and times for the electronic populations are indicated by black arrows in Fig S1, with the notation τ_{ij} that is the overall relaxation time from subband i to subband j. Relaxation rates for coherences ρ_{ij} are noted γ_{ji}; the density matrix equations can thus be written:

(5) $\frac{\partial \rho_{ij}}{\partial t} = \frac{1}{i\hbar} \langle [\sum_{\boldsymbol{k}} c_{i\boldsymbol{k}}^{\dagger} c_{j\boldsymbol{k}} , \hat{H}] \rangle - \gamma_{ji}\rho_{ij}$, $\frac{\partial N_i}{\partial t} = \frac{1}{i\hbar} \langle [\sum_{\boldsymbol{k}} c_{i\boldsymbol{k}}^{\dagger} c_{i\boldsymbol{k}} , \hat{H}] \rangle - N_i \sum_{j\neq i} \frac{1}{\tau_{ij}} + \sum_{j\neq i} N_j/\tau_{ji}$

For the photonic degrees of freedom, we apply a similar approach that is reminiscent of input-output relations. To this end, we introduce coupling between the cavity and an external incoming S_{in} and reflected field S_{out} and we introduce coefficients that describe the radiation Γ_r and non-radiation loss in the system Γ_{nr}. To express the equation of motion we project the cavity fields into a coherent state $a|\alpha> = \alpha|\alpha>$, and we introduce the two quadratures of the field $A = \alpha + \alpha^*$ and $E = i(\alpha^* - \alpha)$. It turns out that for the complete set of equation it is also convenient to introduce the quadratures for the coherences of the density matrix:

(6) $S_{ij} = \rho_{ij} + \rho_{ji}, \quad D_{ij} = i(\rho_{ij} - \rho_{ji})$

Which is a real quantity since $\rho_{ij} = \rho_{ji}^*$. We further introduce the following useful quantities:

(7) $I = \Omega_{R1}E + \chi S_{12}, \quad \chi = \frac{\omega_{P1}^2}{2\omega_{21}}$

(8) $N_0 = N_1 + N_2 + N_3 + N_4 + N_5$

Here N_0 is the total number of electrons in the structure. We note that at equilibrium and at T=0 K the Fermi level E_F lies just below the second subband.

Assuming zero bias applied on the structure, the full set of evolution equations is:

(9) $\frac{dN_1}{dt} = \frac{N_5}{\tau_{51}} + \frac{N_2}{\tau_{21}} - ID_{12}$

(10) $\frac{dN_2}{dt} = \frac{N_3}{\tau_{32}} - \frac{N_2}{\tau_{21}} + ID_{12} - \Omega_t D_{23}$

(11) $\frac{dN_3}{dt} = -N_3\left(\frac{1}{\tau_{32}} + \frac{1}{\tau_{34}}\right) + \Omega_t D_{23}$

(14) $\frac{dN_4}{dt} = \frac{N_3}{\tau_{34}} - \frac{N_4}{\tau_{45}}$

(15) $\frac{dN_5}{dt} = \frac{N_4}{\tau_{45}} - \frac{N_5}{\tau_{51}}$

(16) $\frac{dS_{23}}{dt} = -\gamma_{32}S_{23} - \omega_{32}D_{23} + ID_{13}$

(17) $\frac{dD_{23}}{dt} = \omega_{32}S_{23} - \gamma_{32}D_{23} + 2\Omega_t(N_2 - N_3) - IS_{13}$

(18) $\frac{dS_{12}}{dt} = -\gamma_{21}S_{12} - \omega_{21}D_{12} - \Omega_t D_{13}$

(19) $\frac{dD_{12}}{dt} = \omega_{21}S_{12} - \gamma_{21}D_{12} + 2I(N_1 - N_2) + \Omega_t S_{13}$

(20) $\frac{dS_{13}}{dt} = -\gamma_{31}S_{13} - \omega_{31}D_{13} - ID_{23} - \Omega_t D_{12}$

(21) $\frac{dD_{13}}{dt} = \omega_{31}S_{13} - \gamma_{31}D_{13} + IS_{23} + \Omega_t S_{12}$

(22) $\frac{dA}{dt} = \omega_c E + 2\Omega_{R1}S_{12}$

(23) $\frac{dE}{dt} = -\omega_c A - 2(\Gamma_r + \Gamma_{nr})E + 2\sqrt{2\Gamma_r}S_{in}$

(24) $S_{out} = -S_{in} + \sqrt{2\Gamma_r}E$

In the above equations we have assumed parabolic band approximation $\omega_{ij} = \omega_{i\mathbf{k}} - \omega_{j\mathbf{k}} = \omega_i - \omega_j$, and we have set $\omega_c = E_{cK=1}/\hbar$. We also assumed a periodic system with N_0 electrons per period, i.e. where the level 5 injects electrons to the level 1' of the next period (see Figure S1). Equations (9)-(21) derive from Eq. (5), assuming the absence of correlations between the photon field and the quantum well coherences: i.e. $\langle(a^\dagger - a)c_{2\boldsymbol{k}}^\dagger c_{1\boldsymbol{k}}\rangle = (\alpha^* - \alpha)\langle c_{2\boldsymbol{k}}^\dagger c_{1\boldsymbol{k}}\rangle$. We call this condition "semiclassical condition"; it should be revised in the deep ultra-strong coupling where one seeks to establish the link between vacuum field fluctuations and dark current of the detector[4]. Eq. (22)-(24) can be seen as a generalization of the usual coupled-mode theory beyond the rotating wave approximation; they are equivalent to the Maxwell's equation to the cavity field coupled with the electronic polarization, with the requirement to satisfy the energy conservation.

The cavity is driven by an incident field that can be written in complex notations as $S_{in}(t) = \mathrm{Re}(\tilde{S}_{in}e^{i\omega t})$. The equations set (7-24) is non-linear and its general solution presents a complex task. However, using simple assumptions we can provide an analytical solution that provides the *constant* photocurrent I_{ph} induced by the incident photon flux $|\tilde{S}_{in}|^2$. The DC photocurrent generation is seen as a rectification effect on the incident harmonic drive[5] $\tilde{S}_{in}e^{i\omega t}$. To obtain the DC response of the system, we assume a first order development of the non-linear set (7-24), where variables can be developed in a Fourier series:

(25) $O(t) = \bar{O} + \mathrm{Re}(\tilde{O}_\omega e^{i\omega t} + \tilde{O}_{2\omega}e^{2i\omega t} + \cdots)$

Here $\bar{O}$ denotes the time average for $O(t)$. We consider the lowest order solution where we keep the first non-zero term in the Fourier series (25) for each variable in the equation set (7-24). This approach is valid if the detector is probed by a weak source and the absorbing transition is far from saturation.

Let us now identify which is the lowest order for the expansion in (25) for each variable of the problem. Clearly, the field variables E and A have zero means values, therefore the lowest order is $\tilde{E}_\omega$ and $\tilde{A}_\omega$. Similarly, eq. (22) indicates that this the case for the coherences S_{12} and D_{12} which correspond to the oscillating dipole that interacts with the cavity. Next, the populations N_i have relaxed to their average values $\overline{N_i}$ in a steady state; it can be shown that the next relevant component is $N_{2\omega,i}$ which leads to non-linear optical effects (this will be discussed in another paper). This assumption is consistent with the form of equations (7-24). For instance, looking at eq. (9) we see that the evolution of N_1 is coupled to the product of two oscillating terms $I \cdot D_{12}$ that will yield a non-zero average. Finally, examining the remaining set of equations we can show that the extractor coherences S_{13} and D_{13} have zero mean value, while the tunneling coherences S_{23} and D_{23} have necessarily non-zero average values $\bar{S}_{23}$ and $\bar{D}_{23}$; those average values allow expressing the 2→3 DC tunneling current in the Kazarinov and Suris approach[6]. In the following, we simplify notations and write the average quantities by O instead of $\bar{O}$ and the complex amplitude of oscillating terms $\tilde{O}$ by instead of $\tilde{O}_\omega$. We also introduce the following scalar product of two oscillating variables:

(26) $\overline{O_1(t)\, O_2(t)} = \frac{\mathrm{Re}(\tilde{O}_1 . \tilde{O}_2^*)}{2} = \langle \tilde{O}_1 \cdot \tilde{O}_2 \rangle$

Next, we set all time derivatives of average terms to zero, and all-time derivatives of oscillating terms to $i\omega\tilde{O}$. We arrive at the following set of algebraic equations, where we have eliminated the populations N_4 and N_5:

(27) $\frac{N_3}{\tau_{34}} + \frac{N_2}{\tau_{21}} = \langle \tilde{I} \cdot \tilde{D}_{12} \rangle$

(28) $N_3 \left(\frac{1}{\tau_{32}} + \frac{1}{\tau_{34}} \right) = \Omega_t D_{23}$

(29) $\gamma_{32} S_{23} + \omega_{32} D_{23} = \langle \tilde{I} \cdot \tilde{D}_{13} \rangle$

(30) $\omega_{32} S_{23} - \gamma_{32} D_{23} + 2\Omega_t (N_2 - N_3) = \langle \tilde{I} \cdot \tilde{S}_{13} \rangle$

(31) $(i\omega + \gamma_{21})\, \tilde{S}_{12} + \omega_{21} \tilde{D}_{12} = -\Omega_t \tilde{D}_{13}$

(32) $-(\omega_{21} + \omega_P^2 / 2\omega_{21}) \tilde{S}_{12} + (i\omega + \gamma_{21})\, \tilde{D}_{12} = 2\Omega_R \sqrt{N_0} \tilde{E} + \Omega_t \tilde{S}_{13}$

(33) $(i\omega + \gamma_{31})\, \tilde{S}_{13} + \omega_{31} \tilde{D}_{13} = -\Omega_t \tilde{D}_{12}$

(34) $-\omega_{31} \tilde{S}_{13} + (i\omega + \gamma_{31})\, \tilde{D}_{13} = \Omega_t \tilde{S}_{12}$

(35) $(i\omega + 2\Gamma_{tot}) \tilde{E} + \omega_c \tilde{A} = 2\sqrt{2\Gamma_r} \tilde{S}_{in}$

(36) $i\omega \tilde{A} = \omega_c \tilde{E} + 2\Omega_R \sqrt{N_0} \tilde{S}_{12}$

Here $\Gamma_{tot} = \Gamma_{nr} + \Gamma_r$. In the above equations, we have introduced the collective plasma frequency ω_P^2 and the collective Rabi frequency Ω_R according to the equations:

(37) $\omega_P^2 = N_0 \omega_{P1}^2 = \frac{N_0 e^2}{m_* \varepsilon \varepsilon_0 S L_{QW}}, \quad \Omega_R = \sqrt{N_0} \Omega_{R1}$

We have supposed that most of the electronic population is on the first subband, $N_0 \gg N_{i>1}$, We have neglected the terms ID_{23} and IS_{23}; this is justified since these terms are negligible as long as saturation

effects are not important. The equation set can then be solved analytically, where all quantities can be expressed as a function of the amplitude of the incident field $\tilde{S}_{in}$.

In a steady state, the electromagnetic energy of the cavity does not vary with time, $\frac{d(|\tilde{E}|^2+|\tilde{A}|^2)}{dt} = 0$. We can thus express the energy conservation of the system, and define the frequency dependent reflectivity $R(\omega)$ and the absorption efficiency $\eta(\omega)$ for the 1→2 transition:

(38) $R(\omega) = 1 - 2\Gamma_{nr}\left|\frac{\tilde{E}}{\tilde{S}_{in}}\right|^2 - \eta(\omega), \qquad \eta(\omega) = -2\Omega_{R1}\frac{\langle \tilde{S}_{12}.\tilde{A}\rangle}{\langle \tilde{S}_{in}.\tilde{S}_{in}\rangle}$

It can be shown that these expressions reduce to the usual coupled-mode theory[7] by performing the rotating wave approximation; however, these expressions are more general and can be applied for an arbitrary light-matter coupling strength, and in the presence of strong collective electronic effects[8]. Finally, the photocurrent is defined as the out-going current $I_{ph} = eN_3/\tau_{34}$ that appears for an incident drive. With this definition, our equations provide a photocurrent that is proportional to the square amplitude of the incident drive, $I_{ph} \propto |\tilde{S}_{in}|^2$. By setting $|\tilde{S}_{in}|^2$ to be the number of photons per second with an energy $\hbar\omega$ we arrive at the following expression for the frequency dependent responsivity of the detector:

(39) $\mathcal{R}(\omega) = \frac{e}{\hbar\omega_{12}}\frac{1}{N_p}G_T G_H(\omega)\eta(\omega).$

Here the factor G_T depends only on scattering times and the tunneling time τ_t, but not on the energy of the incident photon:

(40) $G_T = \frac{1}{1+\tau_{34}\frac{\tau_t}{\tau_{21}}\left(\frac{1}{\tau_{32}}+\frac{1}{\tau_{34}}+\frac{1}{\tau_t}\right)}, \quad \frac{1}{\tau_t} = \frac{2\Omega_t^2\gamma_{32}}{\omega_{32}^2+\gamma_{32}^2}$

We can thus express Eq. (40) as $G_T = 1/(1+\tau_t/\tau_{eff})$ where $\tau_{eff}^{-1} = \frac{\tau_{34}}{\tau_{21}}\left(\frac{1}{\tau_{32}}+\frac{1}{\tau_{34}}+\frac{1}{\tau_t}\right)$, as stated in the main text. Since $0<G_T<1$ we can relate G_T to the extraction probability p_e previously introduced for QCDs [9,10]. The factor $1/N_p$ is commented further.

The absorption quantum efficiency from Eq.(38) depends on the mean value $\eta(\omega) \propto \langle \tilde{S}_{12}.\tilde{A}\rangle = \langle(\rho_{12}+\rho_{21}).\tilde{A}\rangle$. Clearly it depends only on the coherence ρ_{12} of the 1→2 transition and its complex conjugate ρ_{21}. Since $\tilde{A}$ is proportional to the intracavity field, we can state, as in the main text, that in essence $\eta(\omega)$ is proportional to the mean value of product of ρ_{12} and the intracavity field. The explicit expression of the absorption efficiency $\eta(\omega)$ is:

(41) $\eta(\omega) = \frac{8F\omega_P^2\omega_c^2\omega\Gamma_r}{|\Pi(\omega)|^2}\,\mathrm{Im}(\eta_s^*\Delta_{21}(\omega))$

Here we have introduced several quantities that appear at intermediate steps of the full calculation. First of all, we define the functions $\Delta_{21}(\omega)$, $\Delta_{31}(\omega)$ and $\Delta_c(\omega)$ which can be seen as the inverse of response functions for the microscopic electronic dipoles and the cavity oscillator:

(42) $\Delta_{21}(\omega) = \tilde{\omega}_{21}^2+\gamma_{21}^2 - \omega^2 + 2i\omega\gamma_{21}, \qquad \tilde{\omega}_{21}^2 = \omega_{21}^2 + \omega_P^2$

(43) $\Delta_{31}(\omega) = \omega_{31}^2+\gamma_{31}^2 - \omega^2 + 2i\omega\gamma_{31},$

(44) $\Delta_c(\omega) = \omega_c^2 - \omega^2 + 2i\omega\Gamma_{tot}.$

In Eq. (42) we introduced the frequency of the intersubband plasmon of the 1→2 transition, which is renormalized owe to collective electronic effects that arise from the third term of the r.h.s. of (1). These effects are negligible for the extractor transition 1→3 with our current quantum design. Next, the algebraic manipulations of the equation set (31-34) lead to a matrix:

(45) $\begin{bmatrix} M_{ss} & M_{sd} \\ M_{ds} & M_{dd} \end{bmatrix} = \begin{bmatrix} 1 & 0 \\ 0 & 1 \end{bmatrix} - \frac{\Omega_t^2}{\Delta_{21}(\omega)\Delta_{31}(\omega)} \begin{bmatrix} \omega_{31} & i\omega + \gamma_{31} \\ -(i\omega + \gamma_{31}) & \omega_{31} \end{bmatrix} \begin{bmatrix} \omega_{21} & i\omega + \gamma_{21} \\ -(i\omega + \gamma_{21}) & \tilde{\omega}_{21}^2/\omega_{21} \end{bmatrix}$

The second term of the r.h.s. of Eq. (45) arises from the connection between the extractor coherences $\tilde{S}_{13}, \tilde{D}_{13}$ and the absorbing coherences $\tilde{S}_{12}, \tilde{D}_{12}$. From the elements of this matrix we further construct two auxiliary dimensionless quantities:

(46) $$\eta_s = \frac{M_{dd} + M_{sd}\frac{i\omega+\gamma_{21}}{\omega_{21}}}{|M|}, \quad \eta_d = \frac{M_{ds} + M_{ss}\frac{i\omega+\gamma_{21}}{\omega_{21}}}{|M|}, \qquad |M| = M_{ss}M_{dd} - M_{sd}M_{ds}$$

Finally, a very important quantity is the denominator $\Pi(\omega)$ in Eq. (41) which is expressed in the following way:

(47) $$\Pi(\omega) = \Delta_{21}(\omega)\Delta_c(\omega) - F\omega_P^2\omega_c^2\eta_s$$

The complex zeroes of this denominator provide the frequencies and damping rates of the light-matter coupled states, while the residues of the function $1/\Pi(\omega)$ is connected to the Hopfield coefficients. This will be discussed in details in a subsequent work. Let us consider a case without tunnel coupling $\Omega_t = 0$ and no damping, $\Gamma_{tot} = \gamma_{21} = 0$. Then Eq. (47) reduces to $\Pi(\omega) = (\tilde{\omega}_{21}^2 - \omega^2)(\omega_c^2 - \omega^2) - F\omega_P^2\omega_c^2$ and the equation $\Pi(\omega) = 0$ corresponds to the polariton equation introduced previously in the bosonic approach[1].

Finally, let us provide the expression of the coherent gain function $G_H(\omega)$:

(48) $$G_H(\omega) = \frac{\omega_{21}}{\omega_c} \frac{\mathrm{Re}\left[\left(\Delta_{21}(\omega) - \eta_s\omega_P^2\right).\left(\eta_d^* - \frac{1}{2\tau_{21}}\xi^*(\omega)\right)\right]}{\mathrm{Im}(\eta_s^*\Delta_{21}(\omega))}$$

Here we have:

(49) $$\xi(\omega) = \frac{1}{\Delta_{31}(\omega)}\left[\omega_{31}(\eta_s + \eta_d Q_{32}) - (i\omega + \gamma_{31})(\eta_d - \eta_s Q_{32})\right], \quad Q_{32} = \frac{\omega_{32}}{\gamma_{32}}.$$

The function $G_H(\omega)$ has been discussed in the main text, and its simplified version and physical meaning is discussed in section 1.3 of the supplementary file. These expressions conclude the analytical solution of the problem for a microcavity coupled quantum detector with tunnel extraction.

Let us now examine the limit of the theory in the absence of extractor coherences $\tilde{S}_{13}, \tilde{D}_{13}$. I that case the matrix (45) becomes an identity and we have $\eta_s = 1$ and $\eta_d = 0$. However, we still keep the coherences D_{23} and S_{23} in order to have tunnel extraction from level 2 to level 3. We call this approach "conventional" detector theory. Alternatively, we can set very strong damping $\gamma_{31} \to \infty$ for the coherences $\tilde{S}_{13}, \tilde{D}_{13}$ which will produce the same effect. In that case, we find the responsivity is provided by the formula:

(50) $$\mathcal{R}(\omega) = \frac{e}{\hbar\omega_{12}} G_T \frac{1}{N_p} \eta(\omega) \times \left\{\frac{\omega_{21}^2 + \gamma_{21}^2 + \omega^2}{2\omega\omega_c}\right\}.$$

The expression in the square brackets is essentially equal to 1 for an optimal detector in the weak coupling regime with $\omega \approx \omega_c \approx \omega_{21}$; we thus recover the well-known result from the literature[9,10,11] .

Let us now comment on the factor $1/N_p$ that appears in Eq.(39) and Eq.(50). In our model, the photocurrent is expressed as $I_{ph} = eN_3/\tau_{34}$, where N_3 is the population on level 3 on a *single period* of the structure. In a permanent regime, the current is continuous across the structure, that is an electron exciting on period to the right (5→1', or 5→right contact in Figure S1) is replaced by an electron entering the period from the left (5''→1, or left contact→1 in Figure S1). This is the "Eulerian" picture of the transport in QCD as described by A. Delga in Ref.[**10**] (page 347). The current I_{ph} is therefore the total

current circulating in the external read-out circuit when the detector is irradiated with a photon flux $|\tilde{S}_{in}|^2$.

In our system of equations we are computing the total absorption of the system, η, and the populations are proportional to η. Thus, for a multiperiod structure, and assuming identical populations N_i of the ith level in all periods, our model actually provides $N_p N_3$ instead of the population N_3 on a single period. Thus we have to correct with a factor $1/N_p$ in order to obtain the correct value of the current I_{ph}.

Another way to justify the factor $1/N_p$ is the expression of the absorption itself from Eq.(40), where it is $\eta(\omega) \propto F\omega_P^2 \propto N_p N_1$; clearly the absorption in our system is proportional to the number of periods N_p and the population of N_1 of a single quantum well in each period. Thus the absorption per QCD period is $\eta(\omega)/N_p$; thus the photocurrent I_{ph} is proportional to $\frac{\eta(\omega)}{N_p}$.

Our approach can be further generalized to more complex structures where several occupied subbands are present. Furthermore, it can be extended to study optical-nonlinearities and saturation in the strong coupling regime. These aspects will be presented in future works.

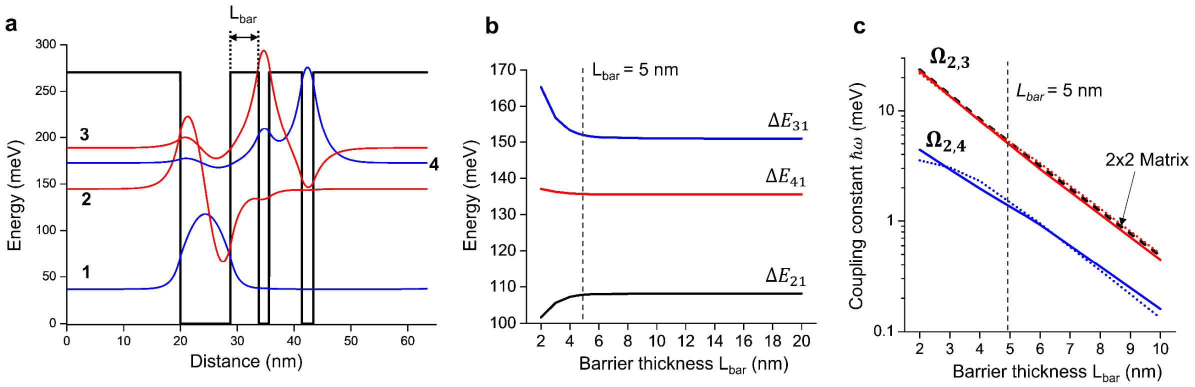

Figure S2. Tunnel coupling strength. a, Bandstructure simulation showing potential and wavefunctions which comprises only the main quantum well and the extractor region. To determine the tunnel coupling one varies the barrier thickness L_{bar}. Charge effect have been neglected for simplicity. **b,** Energy differences $\Delta E_{i1}=E_i-E_1$ as a function of the barrier thickness L_{bar}. (c) Coupling constants obtained from various approaches as described in the text.

1.2. Tunnel coupling and equations in the extended basis

Let us consider only the tunneling Hamiltonian for levels 2 and 3:

$$(51)\quad \hat{H}_t = \sum_{\mathbf{k}} \hbar\omega_{3\mathbf{k}} c^\dagger_{3\mathbf{k}} c_{3\mathbf{k}} + \sum_{\mathbf{k}} \hbar\omega_{2\mathbf{k}} c^\dagger_{2\mathbf{k}} c_{2\mathbf{k}} + \hbar\Omega_t \sum_{\mathbf{k}} \left(c^\dagger_{2\mathbf{k}} c_{3\mathbf{k}} + c^\dagger_{3\mathbf{k}} c_{2\mathbf{k}}\right)$$

This single particle Hamiltonian can be cast in a matrix form:

$$(52)\quad \hat{H}_t/\hbar = \sum_{\mathbf{k}} \left(c^\dagger_{3\mathbf{k}}, c^\dagger_{2\mathbf{k}}\right) \begin{pmatrix} \omega_{3\mathbf{k}} & \Omega_t \\ \Omega_t & \omega_{2\mathbf{k}} \end{pmatrix} \begin{pmatrix} c_{3\mathbf{k}} \\ c_{2\mathbf{k}} \end{pmatrix}$$

We can diagonalize this Hamiltonian by diagonalizing the 2x2 matrix defined above:

$$(53)\quad \begin{pmatrix} \omega_{3\mathbf{k}} & \Omega_t \\ \Omega_t & \omega_{2\mathbf{k}} \end{pmatrix} = U_{\mathbf{k}}^{-1} \begin{pmatrix} \omega_{b\mathbf{k}} & 0 \\ 0 & \omega_{a\mathbf{k}} \end{pmatrix} U_{\mathbf{k}}, \quad U_{\mathbf{k}} = \begin{pmatrix} u_{\mathbf{k}} & v_{\mathbf{k}} \\ -v_{\mathbf{k}} & u_{\mathbf{k}} \end{pmatrix}$$

This procedure defines a Hopfield transform for two new states (b,a) with eigenenergies:

$$(54)\quad \begin{pmatrix} c_{b\mathbf{k}} \\ c_{a\mathbf{k}} \end{pmatrix} = U_{\mathbf{k}} \begin{pmatrix} c_{3\mathbf{k}} \\ c_{2\mathbf{k}} \end{pmatrix}, \quad u_{\mathbf{k}} = \frac{\Omega_t}{\sqrt{\Omega_t^2 + (\omega_{b\mathbf{k}} - \omega_{3\mathbf{k}})(\omega_{2\mathbf{k}} - \omega_{a\mathbf{k}})}}, \; v_{\mathbf{k}} = -\frac{\omega_{b\mathbf{k}} - \omega_{3\mathbf{k}}}{\sqrt{\Omega_t^2 + (\omega_{b\mathbf{k}} - \omega_{3\mathbf{k}})(\omega_{2\mathbf{k}} - \omega_{a\mathbf{k}})}}.$$

(55) $\omega_{b,a\mathbf{k}} = \frac{1}{2}\left(\omega_{3\mathbf{k}} + \omega_{2\mathbf{k}} \pm \sqrt{(\omega_{3\mathbf{k}} - \omega_{2\mathbf{k}})^2 + 4\Omega_t^2}\right)$

The matrix element of the transformation matrix has been normalized so that the fermionic commutation rules hold for the new operators.

We have determined the tunnel coupling energy $\hbar\Omega_t$ for our structure by simulating the main quantum well and the extraction region for various values of the tunnel barrier L_{bar}, as indicated in Figure S2(a).

In Figure S2(b) we plot the energy differences $\Delta E_{i1} = \hbar\omega_{i\mathbf{k}} - \hbar\omega_{1\mathbf{k}}$ as a function of L_{bar}. In the 2x2 matrix approach described above we can infer the coupling constant from the following equation:

(56) $4\hbar^2\Omega_t^2 = \left(\Delta E_{31}(L_{bar}) - \Delta E_{21}(L_{bar})\right)^2 - \left(\Delta E_{31}(L_{bar} \to \infty) - \Delta E_{21}(L_{bar} \to \infty)\right)^2$

Which follows directly from Eq.(54), as we expect that for very large values for L_{qw} we recover the uncoupled energies. The result has been plotted in Figure S2(c) in black dashed curve; this approach yields a value $\hbar\Omega_t = \hbar\Omega_{2,3} = 5.1$ meV for L_{bar} =5nm.

From Figure S2 (b) we see that the ΔE_{i1} is affected as well from the tunnel coupling; in order to estimate that effect we have analyzed the energies with a 3x3 matrix of the form:

(57) $\begin{pmatrix} \Delta E_{41} & 0 & \hbar\Omega_{2,4} \\ 0 & \Delta E_{31} & \hbar\Omega_{2,3} \\ \hbar\Omega_{2,4} & \hbar\Omega_{2,3} & \Delta E_{21} \end{pmatrix}$

The corresponding coupling constants are plotted as continuous lines in Figure S2(c); the corresponding values are $\hbar\Omega_{2,3} = 4.9$ meV, $\hbar\Omega_{2,4} = 1.3$ meV and for L_{bar} =5nm. Therefore, in our model we have privileged the tunnel coupling between levels 2 and 3. Finally, these values have been doubled-check against the predictions of the transfer Hamiltonian approach[12] , where the coupling constant is expressed as:

(58) $\hbar\Omega_t = \frac{\hbar^2}{2m*}\left(\psi_2 \frac{\partial\psi_3}{\partial z} - \psi_3 \frac{\partial\psi_2}{\partial z}\right)\Big|_{z=a}$

Here the probability current density is computed at the barrier. The results from this expression are plotted as dotted curves in Figure S2(c) and they corroborate the values from the previous estimations: $\hbar\Omega_{2,3} = 5.1$ meV, $\hbar\Omega_{2,4} = 1.5$ meV.

The equation of motion described in 1.1. can also be written in the extended basis described above. In the resulting equation are equivalent to the description in 1.1., but appear in a slightly more complicated form. More general set of equations, which feature an optical transition between subbands 1 and 3 and plasmon-plasmon couplings between 1→2 and 1→3 will be discussed elsewhere.

1.3. Discussion of $G_H(\omega)$ and a simplified expression

In our model, the photocurrent is expressed from the population of the extractor level N_3. It is therefore interesting to examine this variable more closely. Using the equation system (27-36) we have the following expression:

(59) $N_3 = \tau_{34}G_t\left\{\langle\tilde{I}\cdot\tilde{D}_{12}\rangle + \frac{\langle\tilde{I}\cdot(\omega_{32}\tilde{D}_{13}+\gamma_{32}\tilde{S}_{13})\rangle}{2\gamma_{32}\Omega_t\tau_{21}}\right\}$

The first contribution, $\langle\tilde{I}\cdot\tilde{D}_{12}\rangle$, is expressed solely from the coherences ρ_{21} and the photon field. The quantity $\langle\tilde{I}\cdot\tilde{D}_{12}\rangle$ is closely linked to the absorption efficiency $\eta(\omega)$; we can show that they are actually proportional to each other, with a factor that depends weakly on the frequency ω. The second contribution is expressed from the "optical rectification" of the coherence ρ_{31} and the optical field. Assuming that $\tilde{D}_{13} \sim \tilde{S}_{13}$ and $\omega_{32} \gg \gamma_{32}$ we can rewrite (59) as

(60) $N_3 \approx \tau_{34} G_t \left\{ \langle \tilde{I} \cdot \widetilde{D}_{12} \rangle + \frac{\omega_{32}}{\gamma_{32}} \frac{\langle \tilde{I} \cdot \widetilde{D}_{13} \rangle}{2\Omega_t \tau_{21}} \right\}$

From this form, we can state that there are essentially two pathways to create a population on level 3 by photon absorption. Th first one is an absorption of a photon between 1→2 and then a transfer from level 2 to level 3; this process is weighted by the tunnel gain factor G_t. This is the process that has been considered so far in the conventional detector theory. Eq.(59) shows a second mechanism, that is linked to the coherence ρ_{31} (the transition 1→3). In the localized basis chosen for our discussion this transition is not directly excited by light. We can however link the coherence ρ_{31} to the optically active coherence ρ_{21} by using the equations of motion (20) and (21). Eliminating $\tilde{S}_{13}$ in favor of $\widetilde{D}_{13}$ the following equation is obtained:

(61) $\frac{d^2 D_{13}}{dt^2} + 2\gamma_{31} \frac{dD_{13}}{dt} + (\omega_{31}^2 + \gamma_{31}^2) D_{13} = \Omega_t \left[\frac{dS_{12}}{dt} - \gamma_{31} S_{12} - \omega_{31} D_{12} \right] \approx -\Omega_t \omega_{31} D_{12}$

The quantity thus satisfies a driven oscillator equation, where the driving term is proportional to the tunnel coupling Ω_t as well as the coherences ρ_{12} (ρ_{21}) and their time derivatives. Eventually, we can simplify the source term once again using the approximation $\widetilde{D}_{13} \sim \tilde{S}_{13}$ and $\omega_{32} \gg \gamma_{32}$, as shown in the last term of Eq.(61). Solving (61) in a rotating wave approximation, we obtain the equality:

(62) $\widetilde{D}_{13} \approx -i\Omega_t \widetilde{D}_{12} \frac{1}{\gamma_{31}} \frac{1}{1 + \frac{(\omega - \omega_{31})^2}{\gamma_{31}^2}}$

It is clear form this expression that second term in Eq.(60) is resonantly enhanced at $\omega \approx \omega_{31}$ and it is proportional to the optical absorption. Eventually, the population N_3 is enhanced by the product $\frac{\eta(\omega)}{1 + \frac{(\omega - \omega_{31})^2}{\gamma_{31}^2}}$; this product is maximized when the extractor transition ω_{31} is matched with the resonance of the optical absorption $\eta(\omega)$, which peaks either at the energy of the collective state in the mesa configuration, or the polariton state in the cavity-coupled detector.

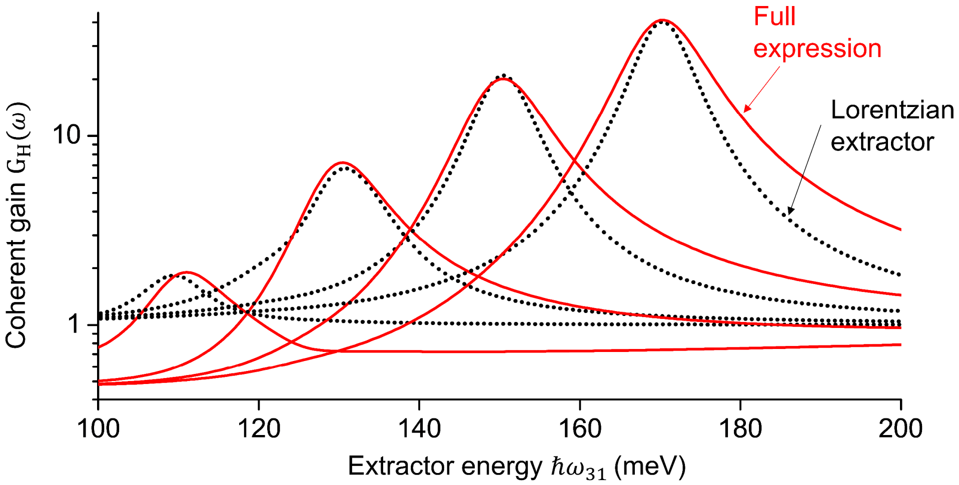

Figure S3. Coherent Gain comparison. Comparison between the full expression for the function $G_H(\omega)$ (full red curves) and the simplified expression from Eq.(59) (black dotted curve).

Inspired from the above discussion, we provide now a simplified expression for the coherent gain function $G_H(\omega)$, specifically for the case of large detuning of the extractor $\omega_{31} \gg \omega_{21}$. A finer approximation is obtained by working directly on the full result from Eq. (48). First, we verify numerically that the parameters in Eq.(46) can be approximated by $\eta_s \approx 1$ and $\eta_d \approx i\eta(\omega)$ where $\eta(\omega)$ is a monotonic function of the frequency with typical values of the order of unity. Next, for sufficiently

high values of ω_{31} the term $\frac{1}{2\tau_{21}}\xi^*(\omega)$ dominates η_d^* on absolute value. We can then provide a simplified expression for $G_H(\omega)$:

(63) $$G_H(\omega) \approx \frac{\omega_{21}}{\omega_c}\frac{(\omega^2-\omega_{21}^2-\gamma_{21}^2)\mathrm{Re}[\xi^*(\omega)\Delta_{31}(\omega))]}{4\tau_{21}\omega\gamma_{21}|\Delta_{31}(\omega)|^2}$$

In the latter expression we have considered the fact that the real part of $\Delta_{21}(\omega)$ dominates the imaginary part for frequencies larger than the resonance ω_{21}. Next, expanding the numerator of Eq.(49) and simplifying the expression for $Q_{32} \gg 1$, at the following simplified expression:

(64) $$G_H(\omega) \approx 1 + \frac{1}{16\omega_c\tau_{21}} \times \frac{\omega_{21}\omega_{31}\omega_{32}}{\gamma_{21}\gamma_{31}\gamma_{32}} \times \frac{\mathrm{Im}(\eta_d)}{1+\frac{(\omega-\omega_{31})^2}{\gamma_{31}{}^2}}$$

Here we have added unity to the simplified expression of $G_H(\omega)$ in order to take into account the first term in Eqs.(59,60). In this simplified version, the function $G_H(\omega)$ appears as a Lorentzian function centered at the extractor transition ω_{31} with an amplitude that grows as the product of the three quality factors $(\omega_{21}/\gamma_{21})(\omega_{31}/\gamma_{31})(\omega_{32}/\gamma_{32})$. In Figure S3 this expression is compared numerically with the full expression of $G_H(\omega)$ for typical values of our detector model. The Lorentzian model reproduces qualitatively the spectral dependence of $G_H(\omega)$ very well; the background value, far from the resonance, is also well accounted from the unity in Eq.(63). In particular the behavior of the maximal value of $G_H(\omega)$ at the extractor energy is well reproduced, especially for the case of strong detuning. This model confirms that the strong increase of the maximum $G_H(\omega = \omega_{31})$ arise from the increasing product $(\omega_{31}/\gamma_{31})(\omega_{32}/\gamma_{32})$. The product of the three frequencies $\omega_{21}\omega_{23}\omega_{31}$ is reminiscent of phenomena encountered in non-linear optics[13]. In the present case it can be argued that the photogeneration of the DC current through light absorption is essentially a rectification phenomenon, where the AC electric field of the incident electromagnetic radiation into a DC current[14]. The non-linearity in this case is provided by the intrinsically non-linear character of the Maxwell-Bloch equations in the fermionic system (Eq.(7-24)), and cannot be recovered from bosonized models. This rectification mechanism, which according to our results is optimized at the extractor frequency ω_{31} allows electron transport at the frequency of the polariton states when ω_{UP} is resonant with ω_{31}.

Supplementary Note 2: Fitting parameters for the quantum model

In the quantum model we used to fit the photocurrent we had to provide the value for radiative and non-radiative losses of the cavity. Since we do not have access to a sample with exactly the same layers and zero doping, we decided to simulate such a device. In particular we simulated the reflectance of a ribbon as a function of its width, i.e. changing the cavity resonance, over a sample characterized by a $\omega_P = 0$. In Figure S4 we report on the left the result of such simulation. From the contrast and width of the resonance we can extract the value of the losses as a function of the cavity frequency:

(65) $C = 1 - R = \left(\frac{4\gamma_r\gamma_{nr}}{\gamma_r+\gamma_{nr}}\right)^2$

(66) $FWHM = \gamma_r + \gamma_{nr}$

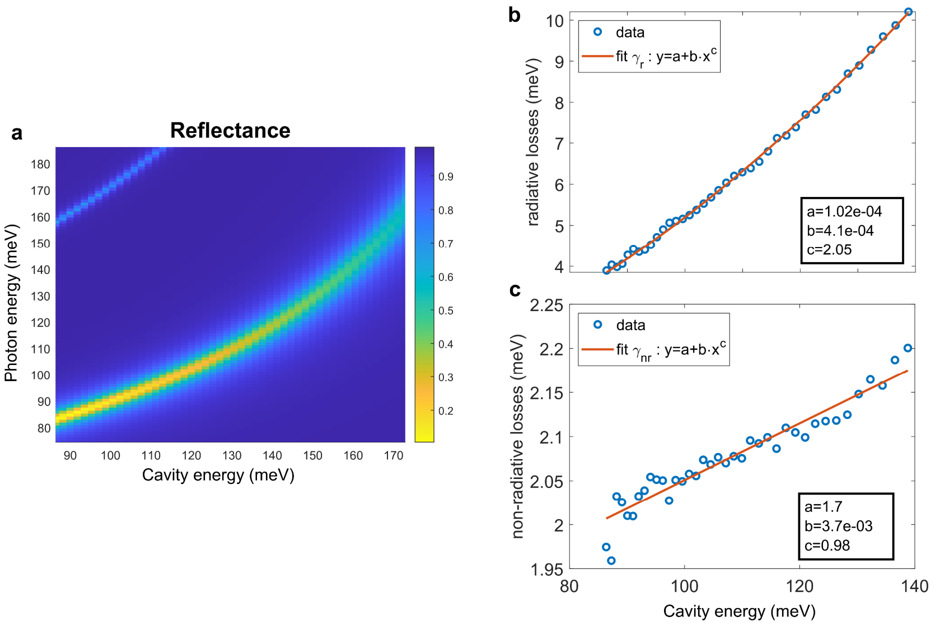

Figure S4. Radiative and non-radiative losses estimation. a Reflectance of the empty cavity simulated with COSMOL Multiphysics as a function of the cavity and photon energy. On the right we reported the radiative (**b**) and non-radiative losses (**c**) extracted from the simulation as function of the cavity energy (dot) and the fitted by a polynomial function (continuous line).

Each value is reported on the right side of figure S4 (blue dots). Both radiative and non-radiative losses where then fitted with a polynomial function (orange line). The results show a linear dependence of the non-radiative losses with the cavity frequency and a quadratic one for the radiative. These functions were used in our quantum model to set the values of the cavity losses for all the device measured.

The QW transition frequency, width and doping, were already discussed in the absorption measurement. The extractor energy ω_{13} was set at 141 meV with a width of $\gamma_{31} = 16\, meV$. The coupling strength Ω_t=4.7 meV, $\gamma_{32} = 2\, meV$, $\tau_{21} = 2\, ps$ and $\tau_{34} = 0.4\, ps$. This last set of parameters was extracted by fitting the data with the model. These values are compatible with the ones reported in the literature [15,16].

Supplementary Note 3: Growth sheet of the device

Devices were obtained by MBE growth. Complete sequence of the grown layers is provided in table S1.

Material	Thickness (Angstrom)	Aluminum percentage	Doping (cm^{-3})
GaAs (Buffer)	2500		
AlGaAs	3000	50%	
GaAs	500		$3 \cdot 10^{18}$
**Start QCD structure: 10 repetitions**			
AlGaAs	50	35%	
GaAs	88		$3 \cdot 10^{18}$
AlGaAs	50	35%	
GaAs	18		
AlGaAs	58	35%	
GaAs	20		
AlGaAs	44	35%	
GaAs	28		
**End QCD structure**			
AlGaAs	40	35	
GaAs	88		$3 \cdot 10^{18}$
AlGaAs	30	35	
GaAs	500		$3 \cdot 10^{18}$

Table S1. Growth sheet of the device investigated in this work. The structure was grown over a GaAs substrate.

Supplementary Note 4: Other characterization of cavity-coupled detectors: Reflectance, Photocurrent temperature and voltage dependence and Responsivity.

4.1. Reflectance

In figure S5a we reported a SEM picture of the photodetectors we fabricated. Each device in the column has different ribbon width w, indicated in microns, and each column is replicated 9 times. The area of photodetection was roughly 100×100 µm^2. The reflectance of each structure is reported in fig S5b. Each spectrum corresponds to the reflection of the TM mode normalized on the TE mode from a grating with roughly 1 mm^2 size. The spectra are stacked on top of each other, from the lowest cavity energy ($w = 2$ µm) to the highest ($w = 1$ µm). The continuous and dotted lines serve as a guide for the eyes representing, respectively, the polaritonic dispersion, the second order cavity mode and the energy of the extraction channel.

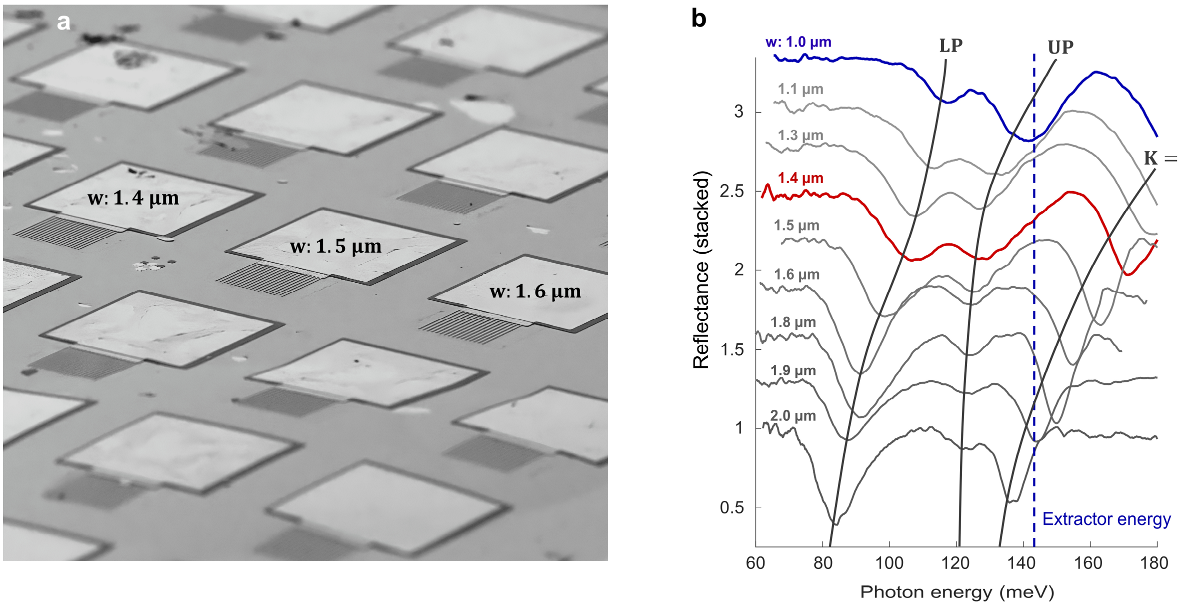

Figure S5. Polariton absorption for different cavity frequencies. a SEM picture of the photodetectors. Each device in the row corresponds to a different ridge width *w* with different cavity energy. The grating area is roughly 100×100 μm^2. **b** Reflectivity spectra TM/TE of 1 mm^2 grating with different ridges width placed above the active region. The lower and upper polariton peaks can be observed for each cavity as well as the second order cavity mode (the black lines are plotted as a guide for the eyes). The data were used to set the parameters of the simulation in Fig.2.

4.2. Photocurrent Temperature dependence

Each device was also characterized in temperature. In Figure S6 (left) we report, in example, the spectrum obtained with a photodetector with ridges width of 1.1 μm (~150 meV) at different temperatures. The photocurrent spectrum can be clearly measured up to 60K with a standard FTIR spectrometer. On the right we report the integral of the photocurrent as a function of the temperature, for different cavities (see also Fig.2(e) of the main text). One can see that the highest photocurrent is measured with ridges width of 1.3 μm. That device is indeed the one with the UP resonant with the extraction channel. The photocurrent signal drops exponentially with the temperature, as the electrons escapes the main QW due to thermal effects.

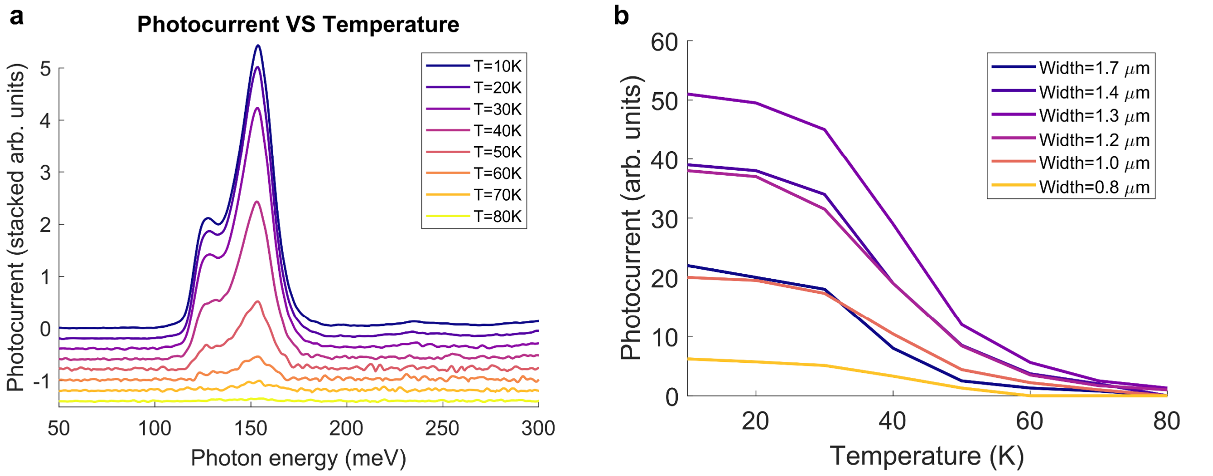

Figure S6. Temperature dependence. The photocurrent measured from the device having ridges with 1.1 μm width is reported on **a** as function of the photon energy at different temperatures (stacked). **b**, the integral of the photocurrent for different cavities is reported as a function of the temperature. The cavity with ridge width of 1.3 μm correspond to the maximum value measured amongst all the devices (fig.2(e) of the main text).

4.3. Photocurrent Voltage dependence

By applying a bias to the detector, we observe a bending of the structure inset in Fig. S7. The energy of the extraction channel changes and so does the spectrum of photocurrent. One can see how the intensity of the photocurrent changes to be maximized by the UP branch at 0 bias to the LP at negative bias. This behavior can be reproduced with our model by changing the energy of the 1→3 transition. In particular by keeping the energy E_{13} as a fitting parameter we managed to reproduce the experimental data with good agreement (right panels of Fig. S7). When applying a positive bias to the structure we observed a more complex behavior: a change of sign in the photocurrent spectra was observed. This suggest the presence of two counter-streaming flows of electrons, one possibly following the extraction channel and the other tunneling above the barrier. Such a phenomenon is not included in our model and for that we limited our comparison to the negative values.

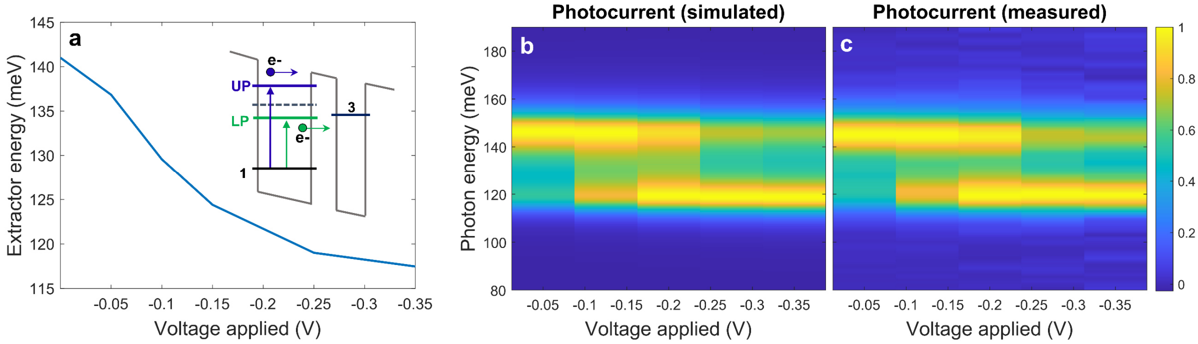

Figure S7. Extractor transition energy as a function of the applied bias. **a,** Estimated extractor energy as a function of the applied bias. The value where founded by fitting the data with our model. In the inset we reported a simplified scheme of the structure showing how the extractor level shift toward the LP energy. Simulated (**b**) and measured (**c**) photocurrent as a function of the applied bias. The model perfectly reproduces the data.

4.4. Responsivity

In order to evaluate the responsivity of our detector at 10K we estimated the power emitted by the glowbar of the FTIR, which is the one of a black body of 1200K, that impinges on the sample. The photocurrent value can be measured with a lock-in, and in particular the value will correspond to the integral of the photocurrent spectra; one can see, e.g. Fig.S6, that all the contribution comes from photons with energy comprised between ~120 and ~175 meV. By dividing the spectral radiance of the glowbar in that range and the integral of the photocurrent we estimated a responsivity of ~50 mA/W. The responsivity of our detectors was also tested with a powerful commercial QCL (MIRcat). The emission of the laser was characterized with a detector as a function of the emitted wavelength, Figure S8 (blue line). Then we shined our device and measured the photocurrent (orange line). The responsivity was calculated assuming that all the power was impinging on our detector, leading to a lower limit of the peak responsivity of ~2 mA/W at 80K. This correspond to ~70 mA/W at 10K, in agreement with the black body measurements.

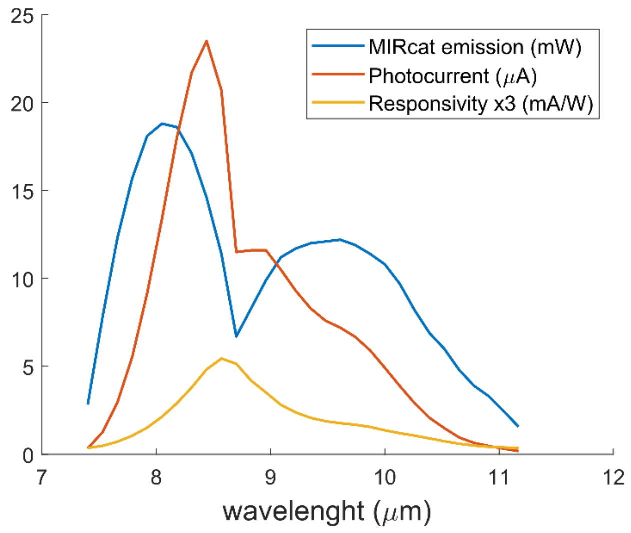

Figure S8. Detector responsivity. Photocurrent (orange line) measured by shining the device with a powerful QCL (MIRcat). By characterizing the MIRcat emission power (blue line) we retrieved the responsivity of our device at 80K (yellow line).

Supplementary References

1. Todorov, Y. & Sirtori, C. Intersubband polaritons in the electrical dipole gauge. *Phys. Rev. B* **85**, 045304 (2012).
2. Todorov, Y. & Sirtori, C. Few-Electron Ultrastrong Light-Matter Coupling in a Quantum LC Circuit. *Phys. Rev. X* **4**, 041031 (2014).
3. Iotti, R. C. & Rossi, F. Microscopic theory of semiconductor-based optoelectronic devices. *Rep. Prog. Phys.* **68**, 2533 (2005).
4. Naudet-Baulieu, C., Bartolo, N., Orso, G. & Ciuti, C. Dark vertical conductance of cavity-embedded semiconductor heterostructures. *New J. Phys.* **21**, 093061 (2019).
5. Dyakonov, M. & Shur, M. Detection, mixing, and frequency multiplication of terahertz radiation by two-dimensional electronic fluid. *IEEE Transactions on Electron Devices* **43**, 380–387 (1996).
6. Kazarinov, R. F. & Suris, R. A. Possibility of the Amplification of Electromagnetic Waves in a Semiconductor with a Superlattice. *Soviet Physics—Semiconductors* 707 (1971).
7. Jeannin, M. *et al.* Absorption Engineering in an Ultrasubwavelength Quantum System. *Nano Lett.* **20**, 4430–4436 (2020).
8. Huppert, S., Vasanelli, A., Pegolotti, G., Todorov, Y. & Sirtori, C. Strong and ultrastrong coupling with free-space radiation. *Phys. Rev. B* **94**, 155418 (2016).

9. Bigioli, A. *et al.* Mixing Properties of Room Temperature Patch-Antenna Receivers in a Mid-Infrared (λ ≈ 9 µm) Heterodyne System. *Laser & Photonics Reviews* **14**, 1900207 (2020).

10. Delga, A. 8 - Quantum cascade detectors: A review. in *Mid-infrared Optoelectronics* (eds. Tournié, E. & Cerutti, L.) 337–377 (Woodhead Publishing, 2020). doi:10.1016/B978-0-08-102709-7.00008-5.

11. Rosencher, E. & Vinter, B. *Optoelectronics*. (Cambridge University Press, 2002). doi:10.1017/CBO9780511754647.

12. Payne, M. C. Transfer Hamiltonian description of resonant tunnelling. *J. Phys. C: Solid State Phys.* **19**, 1145 (1986).

13. Handbook of Nonlinear Optics. *Routledge & CRC Press* https://www.routledge.com/Handbook-of-Nonlinear-Optics/Sutherland/p/book/9780824742430.

14. Unterrainer, K. Chapter 3 Photon-Assisted Tunneling in Semiconductor Quantum Structures. in *Semiconductors and Semimetals* (eds. Liu, H. C. & Capasso, F.) vol. 66 127–186 (Elsevier, 1999).

15. Luo, T., Garg, J., Shiomi, J., Esfarjani, K. & Chen, G. Gallium arsenide thermal conductivity and optical phonon relaxation times from first-principles calculations. *EPL* **101**, 16001 (2013).

16. Lee, N.-E., Zhou, J.-J., Chen, H.-Y. & Bernardi, M. Ab initio electron-two-phonon scattering in GaAs from next-to-leading order perturbation theory. *Nat Commun* **11**, 1607 (2020).
